# LncRNA in gastric cancer drug resistance: deciphering the therapeutic strategies

**DOI:** 10.3389/fonc.2025.1552773

**Published:** 2025-04-01

**Authors:** WeiChi Liu, WeiFa Wang

**Affiliations:** Department of Gastrointestinal Surgery, Chengdu Seventh People’s Hospital, Chengdu, Sichuan, China

**Keywords:** lncRNA, gastric cancer, drug resistance, therapy, precision medicine

## Abstract

Gastric cancer (GC) is an exceedingly aggressive disease and ranks as the third leading cause of cancer-related deaths, which poses a huge health burden globally. Chemotherapy is commonly employed during the middle to advanced stages of cancer, although it faces frequent treatment failures attributed to drug resistance. Thus, it is imperative for researchers to identify potential targets for overcoming therapeutic resistance, thereby facilitating the development of novel anti-cancer agents for GC patients with advanced stages. Long noncoding RNAs (lncRNAs) are a diverse group of transcripts with limited protein-coding capacity, which have been recognized for functional molecules for regulating cancer progression including cell proliferation, metastasis, and drug resistance in GC. In this review, we examine the intricate molecular networks on the role of lncRNAs in drug resistance of GC. LncRNAs conferred cancer cell resistance to anti-cancer drug through various molecular mechanisms, therefore functioning as promising therapeutic targets for GC patients. Additionally, we discuss current advancements of strategies targeting lncRNAs in cancer therapy, which may pave the way for lncRNA-mediated precision medicine for this malignant disease.

## 1 Introduction

Gastric cancer (GC) remains the second most prevalent types of gastrointestinal cancers and ranks the fifth of cancer-related deaths globally in 2022, which significantly contributes to estimated 6.8% of the overall cancer mortality rate (1). Risk factors of gastric cancer include infection with Helicobacter pylori, advancing age, unhealthy lifestyle and diets (2). Nowadays, the disease diagnosis is made histologically following an endoscopic biopsy, and staging is performed using imaging examinations such as laparoscopy, CT scans, and endoscopic ultrasound (3). It exhibits significant molecular and phenotypic heterogeneity, which poses challenges for disease diagnosis and treatment (2). Currently, the primary treatment for early-stage GC is implicated in the surgical resection (4). But due to concealed symptoms in the tumorigenesis, most patients are diagnosed at advanced stages with distant metastasis, who are no longer a suitable candidate for surgical treatment (5). Therefore, chemotherapy, radiotherapy, molecular targeted therapy, immunotherapy or a combination of these methods function as feasible options for advanced GC patients with distant metastasis (5). Although tumors enter remission rapidly under standard therapy, they develop resistance, ultimately leading to treatment failure and disease relapse (6). Therefore, it is essential for researchers to identify potential targets for overcoming therapeutic resistance and pave the way for the developing novel anti-cancer agents in advanced GC.

Long noncoding RNAs (lncRNAs) are a diverse group of transcripts with limited protein-coding ability that exceed 200 nucleotides in length (7). Initially, lncRNAs were considered as “noise” produced during the RNA transcription process due to the lack of protein-coding capability (8). However, with ongoing research, lncRNAs have been found to possess limited protein-coding potential and play significant roles in various pathophysiological processes through complex molecular mechanisms (9–11). Dysregulated lncRNAs function as oncogenes or tumor suppressors that regulate cell proliferation, invasion, migration, metabolic reprogramming, and therapeutic resistance across multiple types of human cancers (12–15). LncRNAs are essential for gene regulation, participating in the regulation of genetic activation and silencing, epigenetic modifications and post-translational regulation, thus impacting tumor progression (15, 16). In the context of GC, abnormal lncRNA expression is closely associated with tumor progression, and therapeutic resistance, thus targeting lncRNAs hold great potential for GC treatment (17–19). However, the detailed molecular mechanisms await further investigations.

In this review, we elaborate the underlying mechanisms involved in the role of lncRNAs in GC progression. Specifically, we depict the intricate mechanistic network of lncRNAs in drug re istance, which indicting that lncRNAs are promising therapeutic targets for GC therapy. Furthermore, we discuss the emerging therapeutic strategies that targeting lncRNAs in cancer therapy, aiming to offer a thorough and structured resource for researchers focusing on lncRNA-based therapeutic therapies.

## 2 Overview of lncRNAs

**Figure 1:**
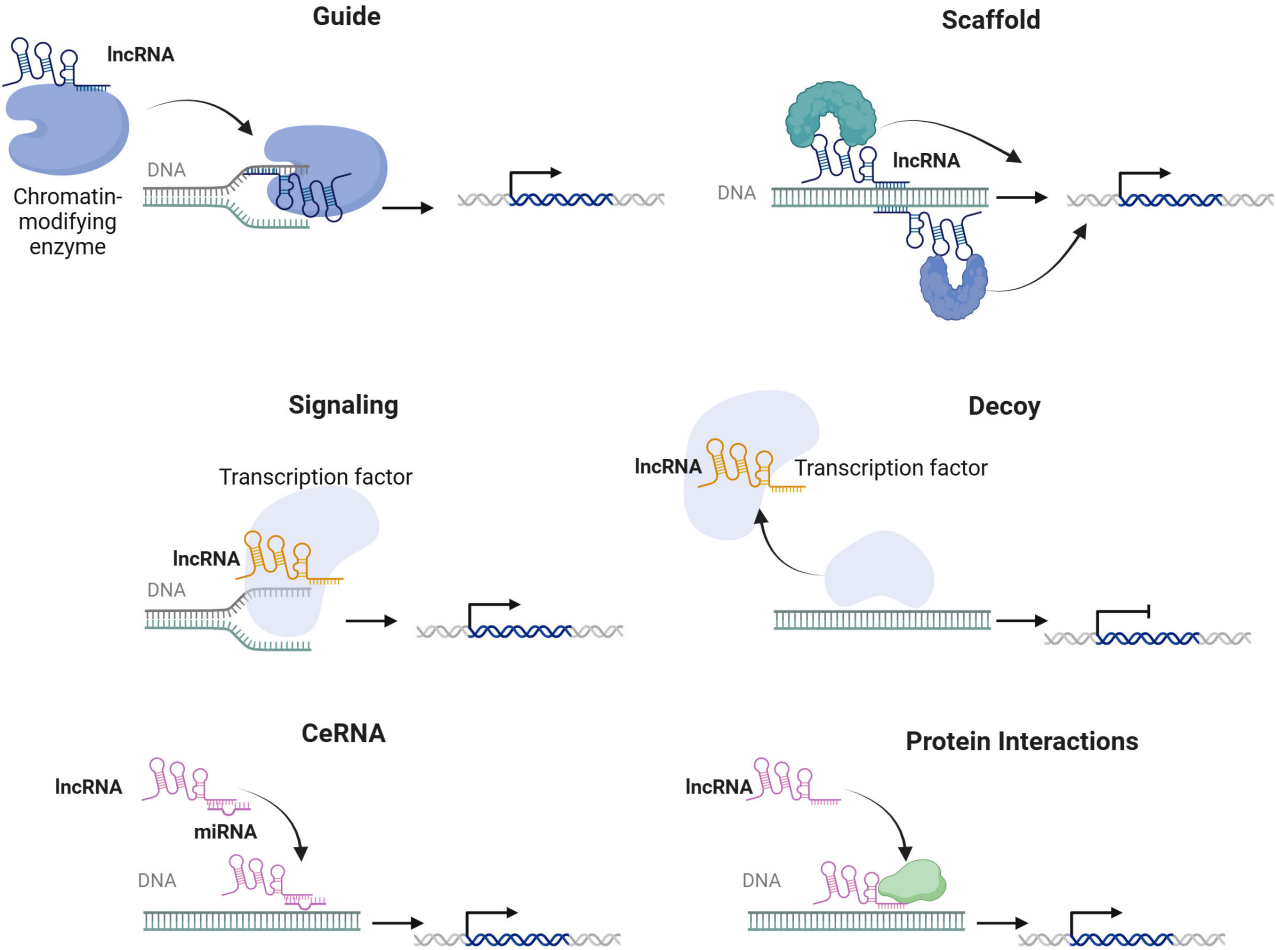
Molecular mechanisms of lncRNAs.

Since the initial identification of lncRNAs through high-throughput sequencing technologies, their extensive potential to modulate the gene expression, transcription, and protein translation has become increasingly evident (20). LncRNAs are synthesized by the activity of RNA polymerase II and display features commonly attributed to protein-coding messenger RNAs (mRNAs), including the presence of a poly-A+ tail and a 5' cap (21). LncRNAs can be classified as intronic, intergenic, sense, antisense, or bidirectional RNAs based on their genomic location, function, and structure (22). This diverse range of genomic locations plays a crucial role in the functional variety and regulatory functions of lncRNAs, which could be present in both the cytoplasm and the nucleus, enabling them to exert their influence and fulfill their functions effectively (23). LncRNAs can also be classified according to their mechanistic modes as signals, decoys, guides, and scaffolds (24). As signals, these RNAs interact with transcription factors in a spatiotemporal manner to regulate gene expression (25). In their decoy role, lncRNAs bind to transcription factors and other proteins, effectively sequestering them from chromatin or directing them into nuclear subdomains (26). Additionally, lncRNAs function as guides by associating with RNA-binding proteins (RBPs) to facilitate their binding to target genes (26). Finally, lncRNAs serve as flexible scaffolds that can accommodate various macromolecules, enable complex formation, and carry out a range of biological functions (27). LncRNAs regulate post-transcriptional gene expression levels in the cytoplasm by stabilizing mRNAs, promoting or inhibiting the translation of target mRNAs through acting as precursors to mRNAs or functioning as competing endogenous RNAs (ceRNAs) by sponging specific target microRNAs (miRNAs) and facilitating mRNA decay (28). In addition, lncRNAs are involved in alternative splicing, the formation of subcellular compartments, and the epigenetic regulation of specific genes (29). Moreover, lncRNAs are found to participate in regulating numerous signaling pathways and targeting multiple downstream target genes (7, 30) (**Figure 1**).

## 3 Molecular mechanisms of lncRNAs-mediated drug resistance in GC

### 3.1 LncRNAs regulate drug resistance by regulating cell apoptosis

Numerous anticancer agents have been identified to trigger apoptosis and engage apoptosis-related signaling pathways (31). Nevertheless, the dysregulation of apoptotic processes frequently contributes to the development of drug resistance and the failure of treatment (31). The regulation of the mitochondrial apoptosis pathway relies on a delicate balance between pro-apoptotic and anti-apoptotic proteins. Disruption of this balance can lead to drug resistance in GC (32, 33). LncRNA plasmacytoma variant translocation 1 (PVT1) induced the expression of anti-apoptotic protein Bcl-2, thus inhibiting cell apoptosis and thereby enhancing the resistance of GC cells to 5-fluorouracil (5-FU) (34). Fang et al. discovered that lncRNA UCA1 sponged miR-27b to promote adriamycin and cisplatin resistance by increase Bcl-2 expression and decreased expression of caspase-3 (35). Small nucleolar RNA host gene (SNHG) encoded a snoRNA and generated a lncRNA, which can regulate gene expression (36). For example, SNHG5 downregulated Bax and upregulated Bcl-2, thereby inhibiting apoptosis and facilitating cisplatin resistance (36).

**Figure 2:**
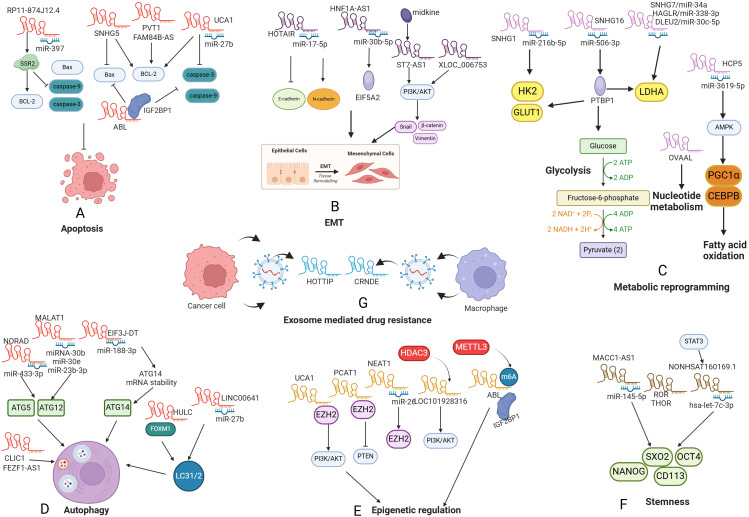
The role of lncRNAs in the drug resistance of gastric cancer. **(A)** LncRNAs promote drug resistance by regulating cell apoptosis; **(B)** LncRNAs promote drug resistance by inducing EMT; **(C)** LncRNAs promote drug resistance by metabolic reprogramming; **(D)** LncRNAs promote drug resistance by promoting cell autophagy; **(E)** LncRNAs promote drug resistance by epigenetic regulation; **(F)** LncRNAs promote drug resistance by promoting cancer stemness; **(G)** Exosomal lncRNAs promote drug resistance.

Caspase family plays an essential role in cell apoptosis and drug resistance. For example, apoptotic protease-activating factor 1 (APAF1)-binding lncRNA (ABL) significantly interacted with the insulin-like growth factor 2 mRNA-binding protein 1 (IGF2BP1), which facilitated the recognition of m6A modifications on ABL, promoting its stability. Additionally, ABL bound to APAF1, consequently blocking apoptosome formation and decreasing the expression caspases-9 and -3. These changes led to multidrug resistance (MDR) in GC (33). Furthermore, RP11-874J12.4 functioned as a molecular sponge for miR-397, which further enhanced the expression of signal sequence receptor subunit 2 (SSR2) that upregulated Bcl-2 expression and downregulated expression of cleaved caspase-3, caspase-9, and Bax, leading to resistance to chemotherapeutic drugs in GC (37). LncRNA FAM84B antisense RNA (FAM84B-AS) augmented cisplatin resistance by suppressing apoptosis via enhancing the expression of BCL-2 and BCL-xL and downregulating the expression of caspase-3, -7, and -9 (38) (**Figure 2**).

### 3.2 LncRNAs induce drug resistance by inducing EMT

Epithelial-mesenchymal transition (EMT) refers to a biological process whereby epithelial cells undergo a phenotypic transformation to acquire mesenchymal characteristics (39, 40). In the context of cancer, EMT is linked to critical metastatic processes such as tumor metastasis and therapeutic resistance (41, 42). Emerging evidence has revealed that lncRNAs regulate GC drug resistance through EMT pathway (43, 44). LncRNAs play a crucial role in mediating EMT-associated drug resistance by modulating EMT markers and transcription factors. LncRNA HOTAIR directly sponged miR-17-5p, leading to downregulation of E-cadherin and upregulation of N-cadherin and Vimentin, thus facilitating both EMT and resistance to multiple drugs including: cisplatin, doxorubicin and 5-FU (45). LncRNA HNF1A-AS1 was reported to be highly expressed in GC tissues, which facilitated cell resistance to 5-FU by promoting miR-30b-5p/EIF5A2 axis-mediated EMT (43). Yang et al. revealed that cancer-associated fibroblast (CAF)-derived midkine (MK) significantly upregulated lncRNA ST7-AS1 in the context of GC, which involved in the cisplatin resistance. Knockdown of ST7-AS1 reversed cisplatin resistance via inhibiting phosphoinositide 3-kinase (PI3K)/AKT pathway and suppressing EMT (44). In line with this, LncRNA XLOC_006753 was found to be upregulated in MDR GC cell lines. The knockdown of XLOC_006753 resulted in decreased levels of Snail, β-catenin, and Vimentin, which ultimately reversed the EMT and enhanced the sensitivity of gastric cancer cells to cisplatin and 5-FU by inactivating PI3K/AKT pathway (46).

### 3.3 LncRNAs induce drug resistance by reprogramming metabolism in GC

Cancer cells undergo metabolic reprogramming to support their survival and cancer progression. This involves heightened glycolytic dependency characterized by increased glucose uptake and fermentation to lactate, satisfying their anabolic requirements for proliferation (47). This phenomenon, known as the Warburg effect, has been reported to induce drug resistance in cancers (48). In the context of GC, emerging evidence has revealed that dysregulated lncRNAs might contribute to drug resistance via regulating glycolysis. Research performed by Xu and colleagues has demonstrated that lncRNA SNHG1 sponged miR-216b-5p and enhanced expression levels of hexokinase 2 (HK2), which functions as a critical enzyme in glycolysis. These changes conferred GC cell resistance to paclitaxel, which provided novel targets for treating chemoresistant patients (49). Furthermore, lncRNA SNHG16 and polypyrimidine Tract Binding Protein 1 (PTBP1) were upregulated in GC cell lines. Mechanistic studies revealed that SNHG16 severed as a ceRNA for miR-506-3p to target PTBP1, which resulting in enhanced mRNA stability of multiple glycolysis enzymes including Glucose Transporter Type 1 (GLUT1), HK2, and lactate dehydrogenase A (LDHA), thus promoted 5-FU resistance, which could be reversed by silencing SNHG16 *in vivo* (50). Bioinformatics analysis revealed that miR-34a has a potential binding site on SNHG7, and this negative relationship was validated in cisplatin-resistant GC tissues. Moreover, LDHA levels could be enhanced by lncRNA SNHG7/miR-34a axis and knockdown of SNHG7 could sensitized cisplatin-resistant GC cells by inhibiting LDHA, suggesting the SNHG7-miR-34a/LDHA-glycolysis axis contributes to cisplatin resistance (51). LncRNA HAGLR was highly expressed in GC cell lines and tissues. It sponged miR-338-3p to target LDHA-mediated glycolysis, thus facilitated 5-FU resistance in the context of GC (52). DLEU2, was another oncogenic lncRNA that targeted LDHA-mediated glycolysis to promote paclitaxel resistance by acting as ceRNA for miR-30c-5p (53). LncRNA HCP5 enhanced fatty acid oxidation via sequestering miR-3619-5p and modulating AMPK/PGC1α/CEBPB pathway, thereby facilitating chemoresistance in GC (54). Recently, Tian et al. reported that lncRNA OVAAL promoted resistance to 5-FU by enhancing pyrimidine biosynthesis, thereby counteracting the thymidylate synthase dysfunction caused by 5-FU (55), indicating that targeting OVAAL-mediated nucleotide metabolic reprogramming presents a promising strategy to overcome 5-FU resistance in GC.

### 3.4 LncRNAs induce drug resistance by regulating autophagy

Autophagy is a conserved cellular degradation process that encapsulates portions of the cytosol and damaged organelles within double-membrane vesicles called autophagosomes (56). Numerous studies have demonstrated that increased levels of autophagy not only improve tumor survival but also enhance drug resistance across various tumor types (56). IL-6 activated autophagy via the IL-6/JAK2 pathway and contributed to chemotherapy resistance (57). In GC, chloride channel 1 (CLIC1) was found to promote the activation of autophagy, thus reducing cellular sensitivity to cisplatin (58). Furthermore, dysregulated lncRNAs were found to induce MDR via targeting autophagy. For example, lncRNA EIF3J-DT was significantly overexpressed in drug-resistant GC cells. EIF3J-DT induced ATG14 expression through directly stabilizing ATG14 mRNA. Additionally, EIF3J-DT prevented the degradation of ATG14 mRNA by sponging miR-188-3p, thus promoting chemotherapeutic resistance (18). Hu et al. demonstrated that lncRNA metastasis-associated lung adenocarcinoma transcript-1 (MALAT1) functioned as a molecular sponge for miR-23b-3p, diminishing its inhibitory effects on the expression of ATG12, which led to the development of autophagy-mediated -MDR in cancer cells to cisplatin and vincristine (59). Researchers have demonstrated that MALAT1 sponged miRNA-30b and miR-30e to target ATG5, thus contributing to cisplatin resistance (60, 61). Furthermore, FEZF1-AS1 modulated MDR of GC cells by regulating ATG5 (62). LncRNA NORAD was an oxidative stress-induced lncRNA that facilitated oxaliplatin resistance by regulating miR-433-3p-ATG5/ATG12 pathway (63), which uncovered a complex interaction between cellular stress and lncRNAs in autophagy-mediated drug resistance.

Additionally, research by Xin et al. has shown that lncRNA HULC interacted with forkhead box M1 (FOXM1), stabilizing this protein, which enhances the B-light chain 3 (LC3)-II/LC3-I ratio and contributes to cisplatin resistance through autophagy. Consistent with these findings, the silencing of HULC has been demonstrated to inhibit autophagy and improve the sensitivity of GC cells to chemotherapy (64). LINC00641 was found to sponge miR-582-5p, activating autophagy flux by evaluating expression of microtubule-associated protein 1A/LC3 I/II and p62, thus conferring cancer cells resistance to oxaliplatin. And silencing LINC00641 could increase the sensitivity of GC cells to oxaliplatin (65).

### 3.5 LncRNAs induce drug resistance by epigenetic regulation

Epigenetic modifications refer to influence gene expression without changing the underlying DNA sequences, which have been extensively explored in tumor progression and drug resistance (66). Enhancer of zeste homolog 2 (EZH2) is responsible for the trimethylation of lysine 27 on histone H3 (H3K27me3), serving an essential role in a Polycomb Repressive Complex 2 (PRC2)-dependent manner, which represses expression of multiple tumor suppressive genes through the H3K27me3 mediated by EZH2 (67). EZH2 was involved in drug resistance across various types of human cancers (68). In the context of GC, lncRNAs induced drug resistance by targeting EZH2. For example, Dai et al. revealed that lncRNA UCA1 was upregulated in GC cells and tissues. Furthermore, UCA1 could recruit EZH2 and promoted PI3K/AKT signaling pathway, which led to cisplatin resistance (69). LncRNA prostate cancer-associated transcript 1 (PCAT1) was upregulated in cisplatin-resistant GC tissues, exerting tumor-promoting effects by interacting with EZH2 and thus epigenetically silencing phosphatase and tensin homolog deleted on chromosome ten (PTEN), leading to enhanced H3K27 trimethylation (70). These changes resulted in GC cell resistance to cisplatin (70). LncRNA NEAT1 sequestered miR-26, which diminished the inhibitory effects on EZH2, which led to oxaliplatin resistance (71). Histone deacetylases (HDACs) plays essential roles in maintaining balance between epigenetic modifications, thus exerting greatly impacts on chemotherapeutic resistance (72). HDAC3 was upregulated in GC cisplatin-resistant cells, which further promoted transcription of lncRNA LOC101928316, leading to the activation of PI3K/Akt/mTOR signaling pathway and enhanced cisplatin resistance (73). m6A methylation is the most prevalent modification found in both mRNA and non-coding RNAs, exerting significant effects on RNA stability, splicing, localization, and translation (74). In MDR-GC cell lines, METTL3 has been shown to elevate the m6A levels of ABL, which interacted with IGF2BP1, a member of the m6A reader family, thus preventing APAF1 from forming apoptotic bodies. This mechanism inhibited apoptosis and contributes to drug resistance (33). These results revealed that lncRNA-mediated epigenetic regulations provide feasible therapeutic targets for overcoming drug resistance in GC.

### 3.6 LncRNAs induce drug resistance by regulating cancer stemness

Cancer stemness is considered a crucial element in tumor development. Cancer stem cells (CSCs) represent a distinct population of cancer cells characterized by their capacity for self-renewal and differentiation, contributing to tumor development and resistance to therapy (75). The stemness markers of CSCs primarily encompass cluster of differentiation 24 (CD24), CD133, SOX2(SRY-box transcription factor 2), SOX9 and c-Myc (76–78). Emerging evidence has revealed that lncRNAs regulating drug resistance via targeting stemness-related markers or pathways. For example, Wang and colleagues reported lncRNA ROR exhibited increased expression in the gastric cancer stem cells (GCSCs), which upregulated multiple stemness-related transcription factors including NANOG, SOX2, CD133 and OCT4. Furthermore, ROR promoted cell resistance to adriamycin and vincristine (79, 80). Zhao et al. revealed that lncRNA NONHSAT160169.1 was upregulated in lapatinib resistant GC cells. Further investigation into the mechanisms by which NONHSAT160169.1 conferred lapatinib resistance revealed that it was induced by the signal transducer and activator of transcription 3 (STAT3) pathway. NONHSAT160169.1 sponged hsa-let-7c-3p and thereby negating its suppressive impacts on the expression of SRY-box transcription factor 2 (SOX2), which suggested a NONHSAT160169.1/hsa-let-7c-3p/SOX2 axis in understanding lapatinib resistance in GC (81). LncRNA THOR mediated the mRNA stability of SOX9, thus enhancing stemness and cisplatin resistance in GC (82). LncRNA MACC1 antisense RNA 1 (MACC1-AS1) acted as a competitive antagonist to miR-145-5p, leading to the upregulation of diacylglycerol cholinephosphotransferase (CPT1) and acetyl-CoA synthetase (ACS), which enhanced expression of stemness markers such as LIN28, SOX2 and OCT4, which contributed to the 5-FU and oxaliplatin resistance (83).

### 3.7 Exosomal lncRNAs in the drug resistance of GC

Exosome plays a vital role in enabling cells to adapt to various internal and external changes involved in processes including injury response and tissue homeostasis. Exosome transfer biological mediators including lncRNA, miRNA, and proteins from donor cells to receipt cells, representing a specific and tightly modulatory communication, which significantly impacts on cancer progression (84). LncRNA CRNDE exhibited highly expression in tumor-associated macrophages (TAMs) in the context of GC. Further mechanistic studies have demonstrated that M2-polarized TAMs secreted exosomes, which transferred CRNDE from TAMs to tumor cells. This significantly enhanced the ubiquitination of PTEN through neural precursor cell expressed developmentally downregulated protein 4-1 (NEDD4-1)-mediated signaling pathways, leading to cisplatin resistance of GC cells (85). Exosomes derived from GC cells packaged lncRNA HOTTIP, which mediated high-mobility group A1 (HMGA1)/miR-218 axis to facilitate cisplatin resistance (86) (**Table 1**).

**Table 1:** The role of lncRNAs in gastric cancer drug resistance.

LncRNA	Mechanism	Function	Drug	Reference
PVT1	Induced the expression of Bcl-2	Inhibited cell apoptosis	5-FU	(34)
UCA1	Sponged miR-27b to increase Bcl-2 expression and decreased expression of caspase-3	Inhibited cell apoptosis	Adriamycin and cisplatin	(35)
SNHG5	Downregulated Bax and upregulated Bcl-2	Inhibited cell apoptosis	Cisplatin	(36)
ABL	Interacted with IGF2BP1, blocked apoptosome formation and decreasing the expression caspases-9 and -3.	Inhibited cell apoptosis	5‐Fu‐ and paclitaxel	(33)
RP11-874J12.4	Sponged miR-397, further enhancing the expression of SSR2 that upregulated Bcl-2 expression and downregulated expression of caspase-3, caspase-9, and Bax,	Inhibited cell apoptosis	Docetaxel and cisplatin	(37)
FAM84B-AS	Enhanced the expression of BCL-2 and BCL-xL and downregulated expression of caspase-3, -7, and -9	Inhibited cell apoptosis	cisplatin	(38)
HOTAIR	Sponged miR-17-5p, leading to downregulation of E-cadherin and upregulation of N-cadherin and Vimentin.	Induced EMT	Cisplatin, doxorubicin and 5-FU	(45)
HNF1A-AS1	Regulated miR-30b-5p/EIF5A2 axis	Induced EMT	5-FU	(43)
ST7-AS1	Activated PI3K.AKT pathway	Induced EMT	Cisplatin	(44)
XLOC_006753	Activated PI3K.AKT pathway	Induced EMT	Cisplatin and 5-FU	(46)
SNHG1	Sponged miR-216b-5p and enhanced expression of HK2.	Enhanced glycolysis	Paclitaxel	(49)
SNHG16	Targeted miR-506-3p/PTBP1 axis and enhanced mRNA stability of GLUT1, HK2, and LDHA.	Enhanced glycolysis	5-FU	(50)
SNHG7	Sponged miR-34a to enhanced expression of/LDHA	Enhanced glycolysis	Cisplatin	(51)
HAGLR	Sponged miR-338-3p to target LDHA	Enhanced glycolysis	5-FU	(52)
DLEU2	Sponged miR-30c-5p to regulate LDHA	Enhanced glycolysis	Paclitaxel	(53)
HCP5	Sequestered miR-3619-5p modulated AMPK/PGC1α/CEBPB pathway	Enhanced fatty acid oxidation	Oxaliplatin and 5-Fu	(54)
OVAAL	Enhanced pyrimidine biosynthesis	Nucleotide metabolic reprogramming	5-FU	(55)
CLIC1	Activation of autophagy	Activation of autophagy	Cisplatin	(58)
EIF3J-DT	Sponged miR-188-3p and promoted ATG14 expression through stabilizing its mRNA	Activation of autophagy	Oxaliplatin and 5-FU	(18)
MALAT1	Sponged miR-23b-3p and increased the expression of ATG12	Activation of autophagy	Cisplatin and vincristine	(59)
MALAT1	Sponged miRNA-30b and miR-30e to target ATG5	Activation of autophagy	Cisplatin	(60, 61)
FEZF1-AS1	Enhanced ATG5 expression	Activation of autophagy	5-FU and cisplatin	(62)
NORAD	Regulated miR-433-3p-ATG5/ATG12 pathway	Activation of autophagy	Oxaliplatin	(63)
HULC	Interacted with FOXM1, enhancing the LC3-II/LC3-I ratio	Activation of autophagy	Cisplatin	(64)
LINC00641	Sponged miR-582-5p, evaluating expression of microtubule-associated protein 1A/LC3 I/II and p62 pathway	Activation of autophagy	Oxaliplatin	(65)
UCA1	Recruited EZH2 and promoted PI3K/AKT signaling pathway	Epigenetic regulation	Cisplatin	(69)
PCAT1	Interacted with EZH2 and epigenetically silenced PTEN.	Epigenetic regulation	Cisplatin	(70)
NEAT1	Sequestered miR-26, diminished the inhibitory effects on EZH2	Epigenetic regulation	Oxaliplatin	(71)
LOC101928316	HDAC3 promoted transcription of LOC101928316, leading to the activation of PI3K/Akt/mTOR signaling pathway	Epigenetic regulation	Cisplatin	(73)
ABL	METTL3 elevated the m6A levels of ABL, which interacted with IGF2BP1, thus preventing APAF1 from forming apoptotic bodies.	Epigenetic regulation	5‐Fu‐ and paclitaxel	(33)
ROR	Upregulated NANOG, SOX2, CD133 and OCT4	Enhanced cancer stemness	Adriamycin and vincristine	(79, 80)
NONHSAT160169.1	STAT3 pathway induced expression of NONHSAT160169.1, which sponged hsa-let-7c-3p and regulated SOX2 expression.	Enhanced cancer stemness	Lapatinib	(81)
THOR	Mediated the mRNA stability of SOX9.	Enhanced cancer stemness	Cisplatin	(82)
MACC1-AS1	Acted as a competitive antagonist to miR-145-5p, upregulated CPT1 and ACS, which enhanced expression of LIN28, SOX2 and OCT4.	Enhanced cancer stemness	5-FU and oxaliplatin	(83)
CRNDE	M2-polarized macrophages secreted exosomes, which transferred CRNDE from macrophages to tumor cells, which enhanced the ubiquitination of PTEN through NEDD4-1-mediated pathways	Exosome	Cisplatin	(85)
HOTTIP	Exosomes derived from cancer cells packaged lncRNA HOTTIP, which mediated HMGA1/miR-218 axis	Exosome	Cisplatin	(86)

## 4 Therapeutic strategies for targeting lncRNAs

**Figure 3:**
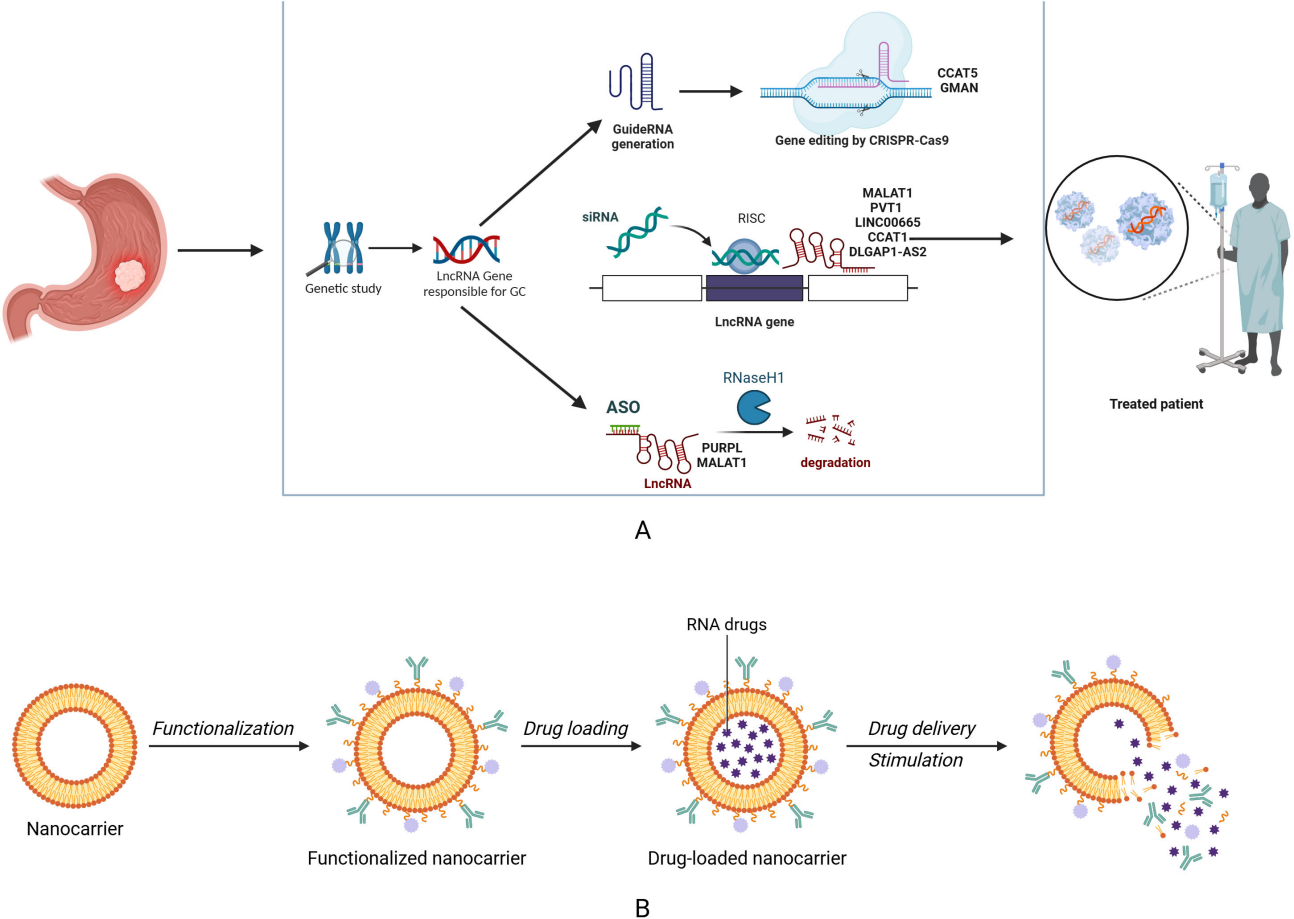
Therapeutic strategies for targeting lncRNAs in cancer therapy. **(A)** CRISPR/Cas 9 technique, antisense oligonucleotides (ASOs), and small interfering RNAs (siRNAs) are capable of degrading lncRNAs, thus providing novel insights into gastric cancer treatment. **(B)** Nanomedicine functions as essential nanocarrier for specifically delivering RNA-based drugs in gastric cancer treatment.

The disruption and function of lncRNAs are progressively being incorporated into cancer treatment strategies, offering benefits in both laboratory and clinical settings (87). The application of RNA-based therapies has emerged as a promising strategy for treating cancers (87). Certain endogenous lncRNAs can modulate the expression of genes associated with tumorigenesis, and their dysregulation may contribute to disease onset, underscoring the potential of these ncRNAs as targets for drug development (87, 88). In GC, lncRNAs can function as either oncogenes or tumor suppressors, leading to the abnormal inhibition or degradation of their target mRNAs. Consequently, they are considered critical therapeutic targets for cancer treatment. In this context, the application of lncRNA-based therapies presents advantages. These emerging strategies included the use of small interfering RNAs (siRNAs), antisense oligonucleotides (ASOs), and clustered regularly interspaced short palindromic repeats (CRISPR)-CRISPR-associated protein (Cas) 9 gene editing (89–91) (**Figure 3**).

### 4.1 siRNAs

SiRNAs are a type of therapeutic agent derived from nucleic acids, functioning as drugs target cytoplasmic RNAs or to induce transcriptional silencing through histone modification and remodeling chromatin within the nucleus by binding to promoter regions (92). This potential has generated considerable excitement in the field of gene therapy. For example, MALAT1 was implicated in GC progression and drug resistance, functioning as a potential target for GC treatment. Experimental assays demonstrated that siRNA-mediated silencing of MALAT1 effectively inhibited the migration and invasion of GC cells (93). Exosomal MALAT1 derived from TAMs was found to enhance chemotherapy resistance. MALAT1 could be transferred from TAMs to cancer cells, while the dual silencing of MALAT1 in tumor cells and TAMs by transferring siRNAs via exosomes significantly enhanced chemosensitivity in the setting of GC (94). Bahar et al. demonstrated that knockdown of PVT1 by specific siRNA could reverse paclitaxel resistance and suppress tumor growth, which suggested a promising approach for GC treatment (95).

### 4.2 ASO

Oligonucleotide technology has been advanced to selectively modify the expression of critical genes involved in terminal illnesses, including human cancers (96). ASOs are chemically modified single-stranded nucleic acid sequences, which could bind complementarily to RNA sequences, thus modulating the functions of mRNA (96). ASOs facilitate the degradation of lncRNAs through the action of RNase H, thereby enabling the silencing and regulation of lncRNAs (97). These characteristics render ASOs a valuable asset in modern medicine, offering high target specificity for treating diseases (98). LncRNA p53-upregulated-regulator-of-p53-levels (PURPL) contributed to chemotherapy resistance, while ASO-based knockdown significantly reduced the expression of PURPL, leading to induced cell apoptosis and sensitized cancer cell to chemotherapeutic agents (99). Furthermore, LNA gapmeR, a specially designed ASO, has found extensive application in preclinical studies. This LNA gapmeR ASO directly induces the degradation of MALAT1, demonstrating remarkable antitumor activity and eliciting cytotoxic effects in a mouse models (100). ASOs that targeted the lncRNA MALAT1 in triple-negative breast cancer has been demonstrated to alleviate malignant characteristics by reshaping tumor microenvironment (101). And ASOs-based silencing of MALAT1 led to reduced cancer metastasis and reversed therapeutic resistance in various types of human cancers (102–104). Given the oncogenic role of MALAT1 in the progression and drug resistance in GC, the suppression of MALAT1 by ASOs hold immense therapeutic potential for GC treatment, which await further investigation both in the preclinical and clinical settings in the context of GC (105–107).

### 4.3 CRISPR/Cas 9

Recent advancements in the CRISPR-Cas9 technique have shown promising results in the precise modification of genes, potentially offering new avenues for cancer treatment (108). Studies employing CRISPR-Cas9 technology have shown encouraging, safe, and effective cancer therapies, marking a significant step forward in the field of precision oncology (109). Emerging preclinical studies have utilized CRISPR- Cas 9 system to target oncogenic lncRNAs, thus inhibiting tumor progression. The knockdowns of RP11-93B14.5, PANDAR, and NEAT1 lncRNAs have demonstrated great effects on the inhibition of cell proliferation, growth, metastasis and therapeutic responses in GC (110–112). In the context of GC, Liu et al. revealed that CRISPR/Cas9-knocknout system-mediated silences of lncRNA CCAT5 significantly impeded tumor growth *in vivo* (113). Moreover, a strategy utilizing CRISPR/Cas9 system to target the lncRNA GMAN led to a significant decrease in the number of metastases generated from GC cells *in vivo* (114). These findings highlight the significant potential of CRISPR-Cas9 technique for translating lncRNA research into clinical applications, which may pave the way for GC treatment.

### 4.4 Nanomedicine-mediated strategies for targeting lncRNAs

Synthetic nanocarriers have long been employed for drug delivery purposes. They have the capacity to encapsulate both hydrophobic and hydrophilic drugs, and their formulations can be modified to enhance stability (115). Emerging evidence has revealed that nanocarriers serve as feasible platforms that can deliver lncRNAs to tumors, potentially enhancing therapeutic results for cancer patients (115). Wang and colleagues reported that lncRNA ABL promoted MDR and tumor growth in GC, and encapsulated liposomal siRNA targeting ABL could markedly increase the sensitivity of GC cells to chemotherapeutic agents (33). The nanocarrier chitosan-gelatin-EGCG (CGE) has been reported to delivery siRNA that targeted TMEM44‐AS1, which significantly abrogated 5-FU resistance in GC (116). The therapeutic delivery of lncRNA LINC00589 via polyethyleneimine-modified mesoporous silica nanoparticles demonstrated significant efficacy in attenuating peritoneal metastasis in GC experimental models (117), offers a promising strategy for enhancing therapeutic efficacy against various malignancies. The capability to modify polymeric nanoparticles facilitates accurate customization of lncRNA delivery systems. However, the intricate nature of these structures presents challenges for maintaining consistent production. Accordingly, progress in polymerization techniques is crucial for enhancing the therapeutic effectiveness of lncRNA-based cancer therapies.

### 4.5 Current evidence in lncRNA-based targeting strategies for GC drug resistance

Existing evidence has revealed that lncRNAs play an essential role in GC drug resistance. Bahar et al. reported that siPVT1 significantly enhanced chemosensitivity to paclitaxel in GC cells (95). Moreover, using siRNA silencing CCAT1 could reversed drug resistance in cisplatin-resistant GC cells by affecting the PI3K/AKT pathway (118). Cells subjected to LINC00665-shRNA treatment exhibited a marked decrease in trastuzumab responsiveness relative to other experimental cohorts (119). The combined application of siRNA-mediated DLGAP1-AS2 knockdown and oxaliplatin significantly enhanced the chemosensitivity of GC cells, reducing the effective dose of oxaliplatin, which markedly induced apoptosis while simultaneously suppressing cell proliferation, and metastatic potential compared to monotherapy (120). Additionally, several studies have revealed that targeting lncRNA NEAT1 (121), NONHSAT160169.1 (81), FUAT1 (122), HOTAIR (123) also reversed drug resistance in GC, providing promising therapeutic strategies in cancer therapy.

## 5 Challenges in targeting lncRNAs for therapeutic applications

Nucleic acid-based therapies have received considerable attention as treatments for a range of diseases, including cancer; nevertheless, caution is necessary in their application. The implementation of nucleic acid-based therapies *in vivo* studies encounters several challenges. Inefficient delivery methods and the low bioavailability of siRNA observed in animal models limit their therapeutic efficacy (124). In addition, ASOs can become trapped within endosomes, which significantly diminishes their bioavailability (125). As a result, it is vital to establish that oligonucleotides have minimal or absent off-target effects (126). Moreover, when utilizing CRISPR/Cas9 technology, it is important to carefully evaluate potential off-target effects, as they could result in unintended consequences (127). Enhancing the stability of these therapies, prolonging their pharmacokinetics, and improving overall drug stability are therefore critical considerations (128). However, given the multiple roles of lncRNAs in various biological activities, thus modulating the expression of specific lncRNAs may trigger a cascade of reactions, potentially resulting in additional side effects during drug application, which await further investigations in clinical settings.

Although nanoparticle-enhanced delivery of lncRNAs holds significant promise in the field of oncology, there are still considerable challenges related to the scalable and cost-effective manufacturing of these nanoscale systems (129). The complexities involved in achieving desired characteristics such as biocompatibility, stability, and targeted specificity necessitate a meticulous design process (130). The intricate regulatory networks involving lncRNAs require additional research to clarify their specific roles and interactions with other molecular pathways. Furthermore, while specific inhibitors targeting lncRNAs have shown promising anti-cancer effects in preclinical studies, their safety and effectiveness in human clinical trials are still uncertain (131). This highlights the need for enhanced targeting strategies and delivery systems to ensure accurate and effective modulation of lncRNAs. In addition, lncRNA-based therapeutic approaches are faced by tolerance issues, particularly immunogenicity, arising from pathogen-associated molecular pattern receptors that recognize RNA structures, which can trigger immune responses (132, 133). Further research should focus on addressing these challenges, which may contribute to the further exploration of cancer treatment strategies.

## 6 Conclusions and future perspectives

LncRNAs represent a class of functional RNA transcripts present in human genomes. Mounting evidence has highlighted the diverse functions and mechanisms through which many lncRNAs operate, increasingly associating them with the onset and progression of cancers. Nonetheless, the exploration of many lncRNAs remains limited. In the context of GC, lncRNAs exert regulatory functions on chromatin remodeling, histone modification, sponging miRNAs, stabilizing mRNA and regulating translation of various proteins, thus significantly impacting cancer progression and drug resistance. Moreover, lncRNAs serve as essential biomarkers for early detection and monitoring of GC. LINC01133 was reported to be decreased in the serum of GC patients. Furthermore, expression of LINC01133 was associated with tumor markers such as CEA and CA19-9 along with various clinicopathological parameters, including tumor size, the tumor-node-metastasis (TNM) stage, and distant metastasis (134), indicating that LINC01133 was an independent prognostic factor for the disease. Research by Jiang et al. has revealed that lncRNA FAM87A served as an independent biomarker for the overall survival of GC patients (135). Further research performed by Liang et al. indicated that the expression levels of lncRNA XIST and ZFPM2-AS1 were significantly elevated in the plasma of GC patients. Additionally, the area under the receiver operating characteristic curve (AUC) was calculated to be 0.62 for ZFPM2-AS1 and 0.68 for XIST, which suggested that these lncRNAs could be used as candidate plasma biomarkers for GC patients (136). Overall, these findings indicate that lncRNAs are associated with various clinical characteristics of GC and could sever as essential biomarkers for early detection and diagnosis of GC. However, a common limitation in these studies is the relatively small sample size. To identify a lncRNA as a potential new biomarker, it is essential to have a sufficiently large study population that can reliably support the candidature of lncRNAs for biomarker development.

Moreover, lncRNAs play an essential role in regulate drug resistance, which includes multiple mechanisms including regulating cell apoptosis, EMT, metabolic reprogramming, autophagy, stemness, and mediating epigenetic regulation. Therefore, targeting lncRNAs may be feasible strategies for overcoming drug resistance and paving the way for GC treatment. Notably, strategies such as siRNAs, ASOs, and CRISPR/Cas 9 technique significantly exhibit remarkable tumor regression effects in the preclinical setting. But there is lacking of investigations on the anti-cancer effects in the clinical settings. Furthermore, concerns have been raised regarding the therapeutic potential of targeting individual lncRNAs, as well as the effectiveness of current targeting strategies, which underscores the necessity for improved targeting strategies and delivery systems to facilitate precise and effective modulation of lncRNAs. Future research should focus on these challenges and promote the translation of current findings into clinical applications, which may improve the prognosis of chemotherapy-resistant GC patients.
